# Decreased accuracy of forensic DNA mixture analysis for groups with lower genetic diversity

**DOI:** 10.1016/j.isci.2024.111067

**Published:** 2024-09-28

**Authors:** Maria Flores, Evan Ho, Cara Ly, Niquo Ceberio, Miguel Guardado, Kamillah Felix, Hannah Mariko Thorner, Matt Paunovich, Chris Godek, Carina Kalaydjian, Rori V. Rohlfs

**Affiliations:** 1 San Francisco State University, Department of Biology, San Francisco, CA 94132, USA; 2 University of California, Los Angeles, Department of Molecular, Cell and Developmental Biology, Los Angeles, CA 90095, USA; 3 University of California, Santa Cruz, Department of Ecology and Evolutionary Biology, Santa Cruz, CA 95060, USA; 4 University of California, San Francisco, Biological and Medical Informatics Graduate Program, San Francisco, CA 94143, USA; 5 George Washington University, Department of Forensic Sciences - Forensic Molecular Biology, Washington, DC 20007, USA; 6 San Francisco State University, Department of Mathematics, San Francisco, CA 94132, USA; 7 University of Oregon, Department of Data Science, Eugene, OR 97403, USA; 8 Yale University, Department of Genetics, New Haven, CT, USA; 9 Northern Arizona University, Department of Applied Physics and Materials Science, Flagstaff, AZ, USA

**Keywords:** Genetics, Genomics

## Abstract

Forensic investigation of DNA samples from multiple contributors has become commonplace. These complex analyses use statistical frameworks accounting for multiple levels of uncertainty in allelic contributions from different individuals, particularly for samples containing few molecules of DNA. These methods have been thoroughly tested along some axes of variation, but less attention has been paid to accuracy across human genetic variation. Here, we quantify the accuracy of DNA mixture analysis over 83 human groups. We find higher false inclusion rates for mixtures with more contributors and for groups with lower genetic diversity. Even for three-contributor mixtures where two contributors are known and the reference group is correctly specified, false inclusion rates are 1e-5 or higher for 36 out of 83 groups. This means that, depending on multiple testing, some false inclusions may be expected. These false positives could be lessened with more selective and conservative use of DNA mixture analysis.

## Highlights

• Groups with lower genetic diversity have higher mixture analysis false positive rates
• Analyses with mis-specified references have somewhat higher false positive rates

## Graphical abstract

**Figure:**
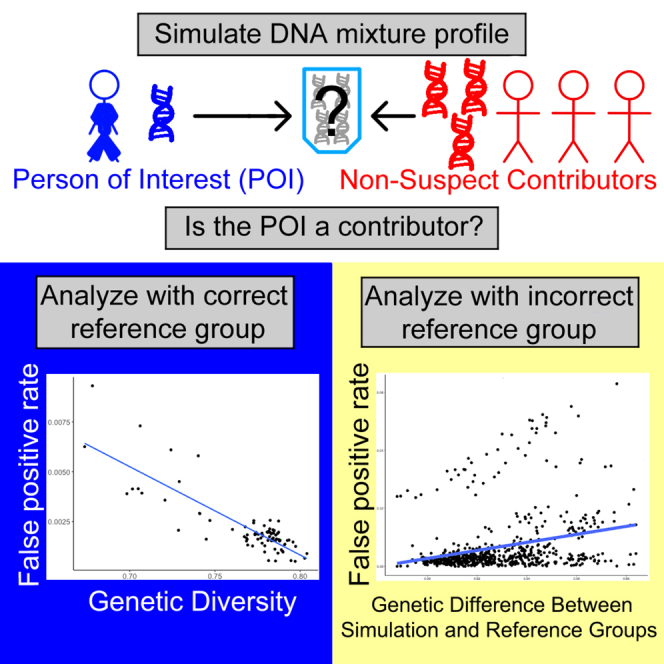

## Introduction

Routine forensic DNA analysis now investigates DNA mixtures (samples with contributions from multiple individuals).^1^^,^^2^^,^^3^ Overlapping alleles between individuals, an unknown number of contributors, stutter, allelic drop-out, and allelic drop-in^4^^,^^5^ complicate DNA mixture analysis.

A number of statistical methods have been developed to analyze such complicated mixture data, including “semi-continuous’ methods based on the presence or absence of alleles, and “continuous” methods that consider peak heights and stutter.^6^ The output of either class of methods is a likelihood ratio (LR) calculated by dividing the probability of observing the mixed DNA profile under a hypothesis that a person of interest (POI) contributed, versus the probability under a hypothesis that the POI did not contribute.^6^ There is significant variability across software packages in the transparency of their methodological approaches, as well as in access to their source code.^7^ Studies have shown that with ideal data, semi-continuous methods approximate continuous methods.^8^

The results of DNA mixture analyses can have a deciding impact on the outcome of an investigation. Therefore, the accuracy of such analyses is of the utmost importance. Accordingly, a large number of studies have investigated the accuracy of DNA mixture analysis.^6^ Particular attention has been paid to developing approaches to validate mixture analysis software.^9^^,^^10^^,^^11^ Studies have investigated accuracy across molecular amounts and ratios,^12^^,^^13^^,^^14^^,^^15^^,^^16^^,^^17^ models (including number of contributors),^17^^,^^18^^,^^19^^,^^20^ and laboratories.^4^^,^^11^^,^^21^^,^^22^

While these studies have defined a scope of reliable DNA mixture analysis under a set of important parameters, they have not considered variability of accuracy along population genetic diversity. Estimation of the frequency of a forensic DNA profile requires reference allele frequency distributions, which themselves are estimated based on a reference group of individuals. For high-quality single-source forensic samples with full genotyping across the Combined DNA Index System (CODIS) loci, estimated profile frequencies may vary by several orders of magnitude depending on the subject’s genetic background and the (possibly mis-specified) reference group, yet the profile frequencies are so miniscule that they still produce decisively informative LRs.^23^^,^^24^^,^^25^^,^^26^^,^^27^^,^^28^ However, in the more complex context of DNA mixture analysis, variation in genetic backgrounds across mixture contributors, POIs, and reference groups may impact the outcome of forensic analyses.

In a different complex forensic technology of familial identification, accuracy is lower for groups with lower genetic diversity, and when allele frequencies are mis-specified.^29^^,^^30^ In DNA mixture analysis, LR calculations are impacted by the reference allele frequencies.^31^ Estimates of the number of contributors to a DNA mixture are less reliable for groups with lower genetic diversity.^32^ Variation in LRs has been observed across broadly defined groups (all with relatively high genetic diversity).^31^^,^^33^^,^^34^ These results all suggest variability in the accuracy of mixture analysis across genetic backgrounds.

Quantification of the accuracy of DNA mixture analysis across realistic human genetic variation is urgently needed as the technique becomes increasingly ubiquitous, raising the probability of coincidental POI inclusions simply due to multiple testing.^12^^,^^35^^,^^36^ We also need to examine mixtures with a higher number of contributors as such samples are increasingly analyzed in case work, particularly in property crime investigations.^12^^,^^36^ Additionally, in casework, the appropriate reference allele frequency distribution is unknown, so we must also estimate the accuracy of DNA mixture analysis under realistic reference allele frequency mis-specification. Typically, analysts perform separate LR calculations with a few reference allele frequency distributions and combine the results in an *ad hoc* procedure.^37^ In some cases, none of these reference groups are representative of the genetic background of the contributors to the mixture.

In this study, we used the free and open source R package, Forensim,^38^ to simulate and evaluate DNA mixtures based on allele frequency distributions from diverse groups. With this approach, we quantify the accuracy, as both false positive rate (FPR) and power, of DNA mixture analysis across groups, including with mis-specified reference groups.

## Results

### Genetic diversity varies across groups

**Figure 1:**
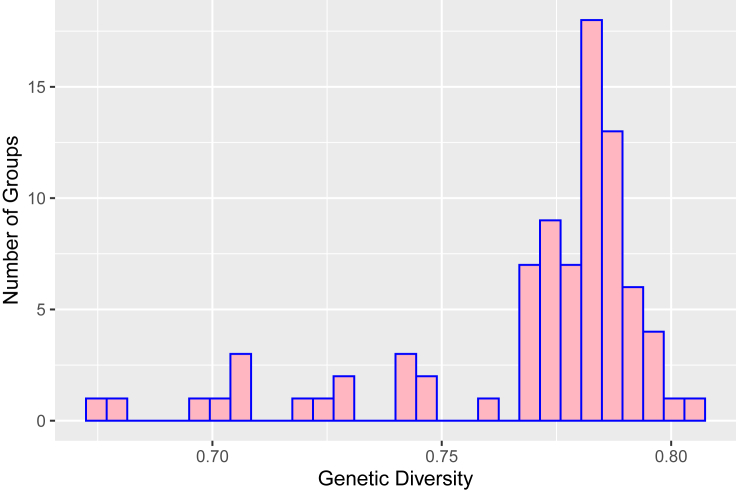
Distribution of genetic diversity over groups Genetic diversity for the 83 groups analyzed for the accuracy of DNA mixture analysis with the correctly specified reference.

Genetic diversity for each group of humans was calculated using allele frequency data obtained from Buckleton et al.’s worldwide survey from 2016, henceforth referred to as “the aggregate study,^39^” (Figures 1 and S1). Genetic diversity, also known as average expected heterozygosity, is $1-\sum_{i}^{n}p_{i}^{2}$ , where *i* is the allele number, *n* is the number of alleles, and *p*_*i*_ is the frequency of that allele. We observe genetic diversity ranging from 0.67 to 0.80 over the 83 groups (Figure 1), and genetic diversity ranging from 0.68 to 0.80 over the subset of 26 groups used for mis-specified reference analysis (see section Accuracy of DNA mixture analysis decreases with mis-specified reference allele frequency distribution) (Figure S1).

### Accuracy of DNA mixture analyses decreases with contributors’ genetic diversity

**Figure 2:**
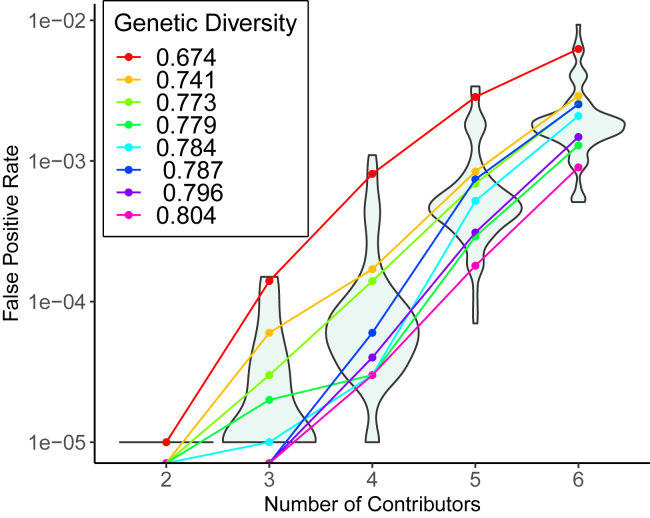
False positive rates when the reference group is correctly specified This plot displays the distributions of false positive rates for two through six contributors from 83 groups. False positive rates are shown for some groups, representing quantiles according to genetic diversity. Note that for two-contributor mixtures 82 groups did not have any observed false positives, so those data points are not plotted with the y axis in log scale. See also Figure S2.

We estimated the accuracy of determining if a POI contributed to a DNA mixture with two through six contributors, over 83 groups, including eight Federal Bureau of Investigation references from the Federal Bureau of Invetigation (FBI). ^40^ In these analyses, we use the correct group as a reference. As expected, FPRs increase with the number of contributors to mixtures (Figures 2, S2, and S3). FPRs as high as 0.0093 were observed for six-contributor mixtures for a group with relatively low genetic diversity (0.68) (Figure 2). Even for mixtures with just three contributors, 17 out of the 83 groups we observe had FPRs above 1e-5 (max of 1.5e-4) (Figure 2).

**Figure 3:**
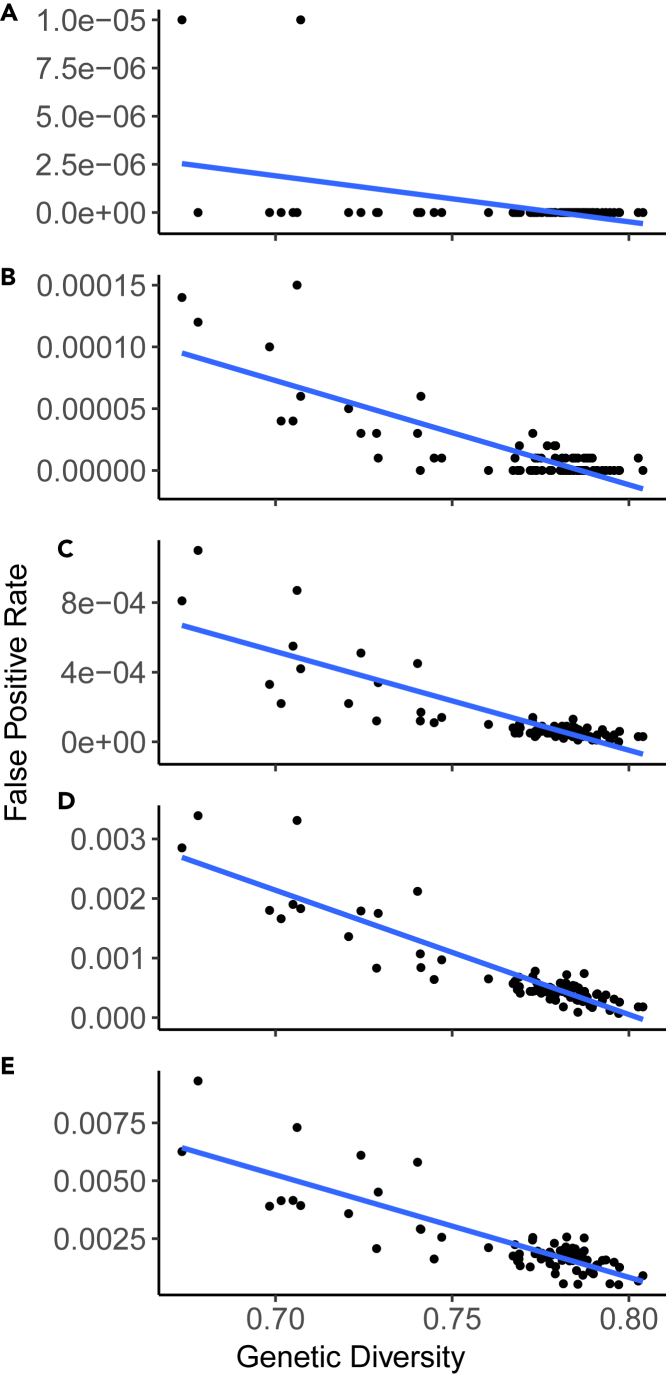
False positive rates versus genetic diversity (A–E) False positive rate versus genetic diversity over groups for mixtures with (A) two contributors, (B) three contributors, (C) four contributors, (D) five contributors, and (E) six contributors. The blue line shows the linear regression between variables.

We observed higher FPRs for groups with lower genetic diversity (r^2^ = −0.44, −0.82,-0.85, −0.91, −0.85, for two through six contributors respectively, *p* = 3.01e-05 for two contributor mixtures, and *p* < 2.2e-16 for three through six contributors Pearson correlation tests) (Figure 3). In other words, the accuracy of DNA mixture analysis decreases when the contributors to the mixture are from groups with comparatively low genetic diversity. The same trends hold with higher likelihood ratio decision thresholds (Figure S6).

Note that when LRs are calculated with higher parameters for the probabilities of dropout, alleles inconsistent with the POI become more easily explained by dropout and the trend of higher FPRs for groups with lower genetic diversity weakens (Figures S4 and S5).

Except for two instances where power was estimated at 0.99993 and 0.99999 for six contributor mixtures (Table S1), we consistently obtained estimations of power equal to 1.0 across groups and number of contributors.

### Accuracy of DNA mixture analysis decreases with mis-specified reference allele frequency distribution

We went on to estimate the accuracy of DNA mixture analysis when the reference group is mis-specified, that is, when the genetic background of the mixture contributors differs from the reference group. For computational efficiency, we used a subset of 26 groups (see STAR Methods section *Subsetting for computational efficiency).*

**Figure 4:**
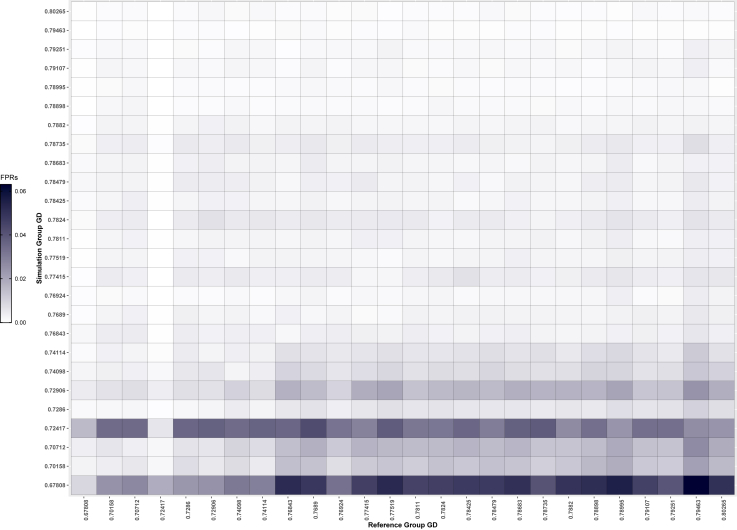
False positive identification rates with correctly and incorrectly specified reference groups The groups are arranged by genetic diversity on each axis. The false positive rates are for the analysis of six-contributor mixtures. Note that the diagonal represents correctly specified reference groups. See also Figure S7.

We find that FPRs vary with simulation group, such that simulation groups with lower genetic diversity tend to have higher FPRs, across correct and mis-specified reference groups (Figure 4). The trend of higher FPRs for groups with lower genetic diversity remains with the FBI reference groups (Figure S7). This is particularly noticeable when there are more contributors in the mixture.

**Figure 5:**
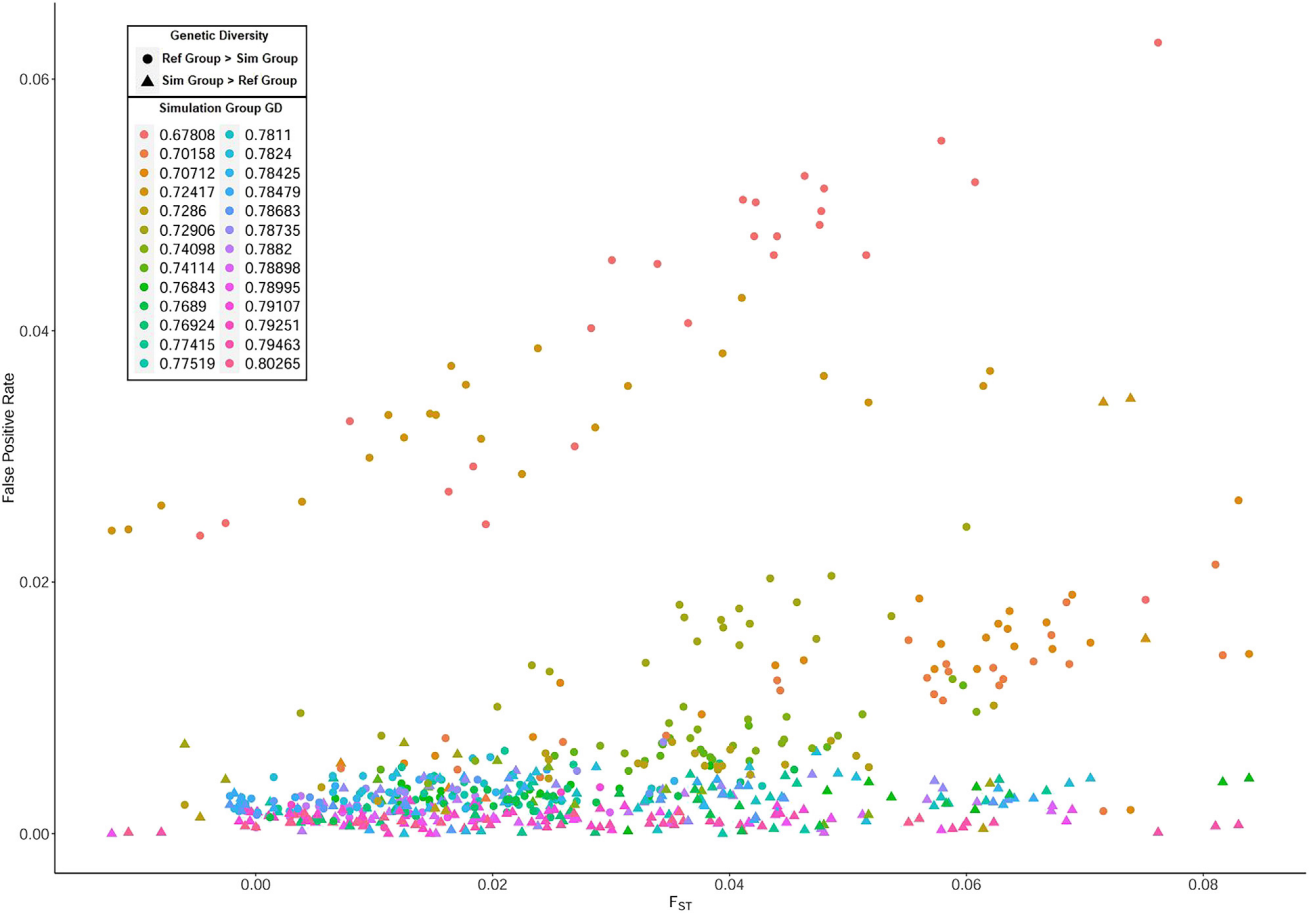
False positive rates versus F_ST_ between the simulation and reference groups False positive rates of six-contributor mixture analyses for each pair of reference and simulation groups are plotted against the F_ST_ between those groups. Each F_ST_ value has two false positive rates because one group serves as the simulation group while the other serves as the reference group, then vice versa. The colors represent the genetic diversity of the simulation group, while the shapes represent whether the reference group or the simulation group has higher genetic diversity.

DNA mixture analysis FPRs vary with incorrectly specified reference groups as well. FPRs are correlated with F_ST_ between the simulation and reference groups (*r*^*2*^ = 0.28, *p* = 5.3e-13, Pearson correlation test) (Figure 5). That is, FPRs are higher as increasingly inappropriate (i.e., genetically distant) reference groups are applied to the mixture analysis. We further see that FPRs are elevated when the mis-specified reference group has higher genetic diversity than the simulation group (Figure 5).

Power remained at 1.0 for all simulations with mis-specified reference groups, with a couple exceptions. There are two groups, genetic diversity 0.724 and genetic diversity 0.6781, that lead to false negatives when they are the reference groups (Figure S8). For those groups, false negatives appear as early as 4 contributor mixtures, and are as high as 0.1473 for a 6 contributor mixture. All instances of false negatives for mis-specified reference group simulations are in Table S2.

Even though power remains at 1.0 for all other simulations, the distribution of LRs varies for analyses of POI+ mixtures. LRs tend to be lower for groups of lower genetic diversity (Figure S9). When the reference group is mis-specified, LRs are inflated (Figures S9 and S10). For Figure S10, we used the second lowest genetic diversity group instead of the lowest genetic diversity group. When we used the lowest genetic diversity group, we found that the LRs were deflated for the high genetic diversity simulation group to low genetic diversity reference group comparison (Figure S11). This was not representative of other mis-specified reference group comparisons, and may be due to the lowest genetic diversity group being an outlier and dissimilar from other low genetic diversity groups. See also Table S2, where the lowest genetic diversity group (along with another group) used as a reference group led to power rates <1 for almost all simulation groups from four through six contributor mixtures. For transparency, we have also included the POI-LRs distribution for the lowest genetic diversity group in Figure S12.

## Discussion

### Discussion of allele frequency distribution data

Our primary source for allele frequency distribution data is Buckleton et al.’s worldwide survey from 2016. This aggregate study^39^ collected data from 250 source papers that quantified the allele frequency distributions for 466 groups. Across (and even within) source papers, these groups were defined based on heterogenous social non-genetic classification schemes including ethnicity^41^^,^^42^^,^^43^, race^44^^,^^45^^,^^46^, nationality^47^^,^^48^^,^^49^, geography^50^^,^^51^^,^^52^, religion^53^, language^54^^,^^55^^,^^56^, tribe^57^^,^^58^^,^^59^, and caste^60^^,^^61^. In many cases, more than one of these frameworks for group identities were described (e.g.: ethnicity, nationality, and geography). Specificity of sampling location also varied from facility^56^, to city^62^^,^^63^^,^^64^, to province/state^65^^,^^66^^,^^67^, and to country^68^^,^^69^^,^^70^. As a result of heterogeneity in classification schemes and specificity of sampling geography, groups described in the source papers have vastly different scales of resolution. Troublingly, samples were also collected from diverse sources including donors who gave informed consent^71^^,^^72^^,^^73^, subjects of paternity tests^74^^,^^75^^,^^76^, people arrested or convicted^77^^,^^78^^,^^79^, collection by law enforcement^58^^,^^80^^,^^81^, immigration casework^82^^,^^83^, and blood banks^84^^,^^85^^,^^86^. The publication of these genetic samples from indirect sources, sometimes without clear informed consent, deserves its own investigation and analysis.^87^ In this study, we limit our analysis to 83 groups where the original publication specified that the sample donors gave informed consent. While standards of informed consent vary,^88^^,^^89^^,^^90^^,^^91^^,^^92^^,^^93^ we hope this limits our re-use of data obtained without consent.

The source studies defined groups based on divergent social constructs across the sociopolitical contexts in which the researchers operated. It is not clear that the individuals sample form homogenous groups, nor that they are representative of the social labels used to describe the groups. These socially constructed groups are not distinct genetic entities or genetic ancestry groups.^87^ While it is abundantly clear that race is a construct created by humans, rather than intrinsically defined by genetic variation,^94^^,^^95^^,^^96^ human genetic research, relevantly in forensics, often relies on models of humans divided into distinct panmictic groups, or admixtures of these panmictic groups.^97^^,^^98^ These models do not reflect the reality of continuous human genetic variation and sustain the invalid idea that race has a biological basis.^99^^,^^100^^,^^101^ Misinterpretation of biological distinction between social groups is worsened when scientists construct, label, and investigate groups based on socially defined characteristics.^102^^,^^103^^,^^104^ The source and aggregate studies apply this social method of grouping and labeling in their reports of allele frequency distributions, in many cases without transparent definition of group descriptors.^105^

While the reported allele frequency distributions would be different if groups were alternatively defined, they do provide insight into one view of the range of variation in CODIS loci allele frequencies across humans. This perspective is useful as a first attempt to explore variation in the accuracy of DNA mixture analysis over empirical human genetic variation. Still, because these groups are based on heterogenous social identities, yet are not obviously representative of those social identities, and do not describe genetic ancestry groups, it would be misleading and counter-productive to label them with those social identities for this genetic analysis.^101^^,^^103^ Since previous analyses found that identification accuracy of another forensic technology (familial searching) is strongly correlated with group genetic diversity, in this study we identify groups by their genetic diversity. For transparency, the previously published group labels and their corresponding genetic diversity are listed in Table S3.

### Discussion of study results

Our results show that DNA mixture analysis has elevated FPRs for groups with lower genetic diversity. FPRs were 1e-5 or higher for 43% of groups, even for two-contributor mixtures where one contributor is known under both the prosecution and defense hypotheses. This is in agreement with previous findings that the reduced identifying information of relatively low genetic diversity groups leads to lower identification accuracy.^30^ We observe higher FPRs correlated with the number of mixture contributors. This elevation is sometimes worsened when LRs are computed with an inappropriate reference allele frequency distribution. In practice the most accurate reference group cannot be known, so elevated FPRs are to be expected, particularly for members of groups with lower genetic diversity.

This analysis does not consider some important variables that arise in DNA mixture analysis in order to focus on our main question of the impact of genetic diversity. While we do not simulate allelic drop in or drop out in our profiles, we included a probability of dropout of 0.01 for heterozygotes and 0.0001 for homozygotes as parameters during LR calculation. Additionally, these analyses are based on simulations and evaluation with no co-ancestry (theta = 0). Accuracy is expected to decrease with realistic non-zero co-ancestry.^106^ Finally, due to computational constraints, we only consider scenarios where non-POI contributors are known under both the defense and prosecution hypotheses. If non-POI contributors are unknown, as is common in casework, we expect accuracy to be lower. These simplifications in our analyses suggest that a more thorough analysis may produce even higher FPRs.

Further studies of the accuracy of DNA mixture analysis are called for to characterize reliability intersectionally over group genetic variation, as well as differences in mixture composition and data quality. Both empirical and simulation-based studies are needed. Such studies would be based on broadly inclusive global estimates of allele frequency distributions gathered with rigorous and consistent procedures for informed consent and data sharing.^107^

As more investigations employ DNA mixture analysis, including in cases involving individuals from groups of relatively low genetic diversity, more false positive identifications will occur, potentially leading to wrongful convictions. This warrants consideration of strategies to prevent these false identifications.

A change in technical approach could decrease the chance of false positive identifications, for example, using a higher co-ancestry coefficient in the LR calculation, as suggested by Steele and Balding.^1^ Still, further investigation is needed to determine the appropriate parameter value for a broader range of groups and for mixtures with more than two contributors. It may be useful to estimate the appropriate reference group(s) and use that in the LR calculations, as suggested for other forensic identification technologies.^108^^,^^109^ However, a single reference group will not accurately reflect genetic backgrounds in mixtures where each individual may have a different most-appropriate group. If implemented, these changes in technical approach should be thoroughly examined for their impact on accuracy, particularly when using popularly applied DNA mixture analysis software.^7^

A different strategy could be to limit the number of cases in which DNA mixture analysis is used. A selective process could filter out DNA mixtures most prone to analytical errors, for example, mixtures with more than three contributors, mixtures where some individuals contribute very little DNA, or mixtures with high estimated drop-out rates. These strategies could be combined with court presentations of the results which include estimates of FPRs.

### Limitations of the study

Our study is sensitive to the categorization schemes used to create groups, including ascertainment biases of what groups were considered, how those groups were defined, and the specific individual-level criteria for inclusion. Because of the opacity and practical limitations of these sampling practices, the resulting published allele frequency datasets imperfectly represent the groups they claim to represent to a certain degree unknowable to us. Thus, we refrain from drawing conclusions about specific social groups based on these results. Since our analysis uses these data, we must be aware that groups defined differently (perhaps by genetic ancestry) would produce different results.

Our study used one particular semi-continuous DNA mixture analysis method. While the trends of the results would likely be similar with other semi-continuous and continuous methods, this study did not specify the specific accuracy with other methods.

## Resource availability

### Lead contact

Further information and requests for resources should be directed to and will be fulfilled by the lead contact Rori Rohlfs (rori@uoregon.edu).

### Materials availability

This study did not generate new unique reagents.

### Data and code availability

• Data: This paper analyzes existing, publicly available data. The links to the datasets are listed in the key resources table.
• Code: All original code has been deposited at https://github.com/Forensic-Mixture-Analysis/ForensicMixtureAnalysis and is publicly available as of the date of publication. DOIs are listed in the key resources table.
• All other requests: Any additional information required to reanalyze the data reported in this paper is available from the lead contact upon request.

## Acknowledgments

We are deeply indebted to the individuals whose genetic information contributed to publicly available data on which this project is based. We appreciate Dr Carolina Adam’s thoughtful comments on the manuscript. This project, particularly R.V.R., M.G., and M.F., was supported by 10.13039/100005289NIJ grant 2019-DU-BX-0028. R.V.R. was supported by 10.13039/100000001NSF CAREER grant 2144878. M.P., M.F., and C.L. were supported by 10.13039/100000002NIH grant T34-GM008574. N.C. was supported by NIH grant R25-GM059298. E.H. and K.F. were supported by the 10.13039/100001127Genentech Foundation Scholars grant #G-7874540. M.G. is a recipient of a Howard Hughes Medical Institute Gilliam Fellowship, Achievement Award for College Scientists Foundation Scholarship, and a UCSF Discovery Fellows Program Award. M.F. is a recipient of the UCLA Eugene V. Cota Robles Fellowship. We gratefully acknowledge that the practices presented in “Equity in author order: a feminist laboratory’s approach” were considered for the authorship of this work.^110^

## Author contributions

Conceptualization, R.R.; methodology, R.R.; software, M.F., E.H., C.L., N.C., M.G., K.F. M.P., C.G., C.K., and R.R.; validation, M.F., E.H., C.L., N.C., M.G., K.F., C.G., and R.R.; formal analysis, M.F., E.H., C.L., K.F., C.G., and R.R.; data curation, M.F., E.H., C.L., N.C., and H.M.T.; writing – original draft, M.F., E.H., N.C., K.F., H.M.T., and R.R.; writing – review and editing, M.F., E.H., N.C., M.G., K.F., H.M.T., and R.R.; visualization, E.H., N.C., K.F., and H.M.T.; supervision, R.R.; project administration, R.R.; funding acquisition, R.R.

## Declaration of interests

The authors declare no competing interests.

## STAR★Methods

### Key resources table

**Table:** 

REAGENT or RESOURCE	SOURCE	IDENTIFIER
**Deposited data**		

Allele frequencies	This paper; Buckleton, J. et al.^46^ and Steffen, C.R. et al.^39^ data	https://github.com/Forensic-Mixture-Analysis/ForensicMixtureAnalysis/tree/main/Allele%20Frequency%20Files https://doi.org/10.1016/j.fsigen.2016.03.004 https://doi.org/10.1016/j.fsigen.2017.08.011

**Software and algorithms**		

R v3.6.1 - *Data Quality Control* R v4.1.3 - *Direct Analysis and Cross Analysis* R v4.2.2 - *Fst Analysis and AEH*	R Core Team^47^	https://www.R-project.org/
Python 3.8.8 -*Data Quality Control*	Python Software Foundation^48^	https://www.python.org/
Forensim	Haned, H.^38^	https://doi.org/10.1016/j.fsigen.2010.03.017 https://forensim.r-forge.r-project.org/
Script to calculate average expected heterozygosity (AEH)	This paper	https://github.com/Forensic-Mixture-Analysis/ForensicMixtureAnalysis/tree/main/AEHFolder
Script to calculate the accuracy of mixture analyses with misspecified reference groups	This paper	https://github.com/Forensic-Mixture-Analysis/ForensicMixtureAnalysis/tree/main/IncorrectReferenceAnalysis
Script to calculate the accuracy of mixture analyses with correctly specified reference groups	This paper	https://github.com/Forensic-Mixture-Analysis/ForensicMixtureAnalysis/tree/main/CorrectReferenceAnalysis
Script to estimate F_st_	This paper; Rohlfs, R. et al.^30^	https://github.com/Forensic-Mixture-Analysis/ForensicMixtureAnalysis/tree/main/FSTFolder
Data Quality Control Scripts	This paper	https://github.com/Forensic-Mixture-Analysis/ForensicMixtureAnalysis/tree/main/GroupQualityControlFolder
Scripts to simulate DNA mixture analysis	This paper	https://github.com/Forensic-Mixture-Analysis/ForensicMixtureAnalysis/tree/main/Simulation_Scripts

### Method details

#### Allele frequency data quality control

**Figure 6:**
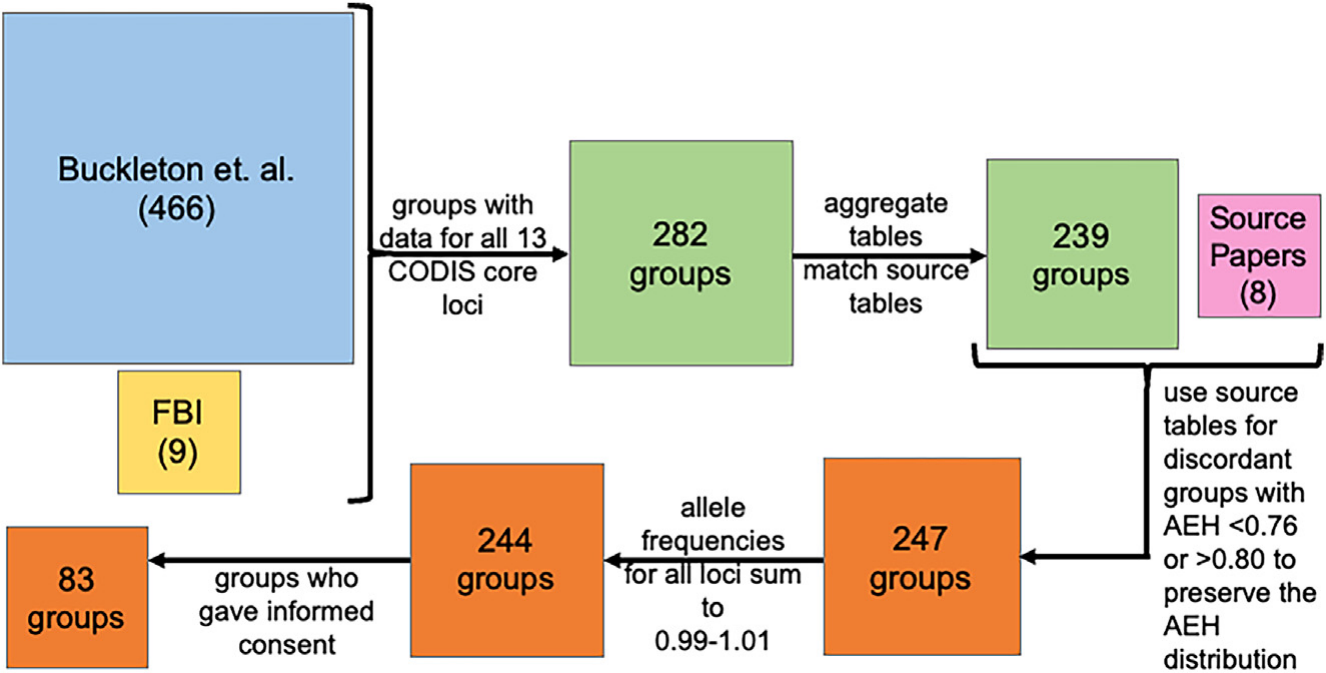
Allele frequency data quality control Boxes reflect the number of groups retained after each quality control step. The analysis started with 475 groups from the aggregate study and the FBI expanded dataset. (1) We only considered groups with allele frequency data for all 13 original core CODIS loci. (2) Because the aggregate study obtained their allele frequency values from other source papers, we only used tables with values that reasonably matched those reported in the source papers. (3) To retain groups representing the full observed range of genetic diversity values, for the groups with unusually high or low genetic diversity values (<0.76 or >0.80), which got removed by the previous filter, we used the source paper data. (4) We then filtered out groups where at least one locus had allele frequencies that did not sum to 1.0 (within 0.01). (5) Finally, we only analyzed the 83 groups where the source paper specified that informed consent was obtained.

Our quality control procedure (Figure 6) aimed to produce a uniform and reliable dataset of allele frequency data for the 13 core CODIS loci across diverse groups. We started with data taken from the previously discussed aggregate study,^39^ as well as the 2015 FBI expanded dataset.^40^ The aggregate study collected data from 250 papers that quantified the allele frequency distributions for 466 groups. With the addition of the FBI dataset, we had 475 distinct groups to consider. First, we considered only the groups that had all 13 original core CODIS loci, leaving us with 282 groups. Second, we cross referenced the allele frequency data reported by the aggregate study^39^ to the cited source papers. We performed the comparison using the 'sdiff’ bash function on.csv files containing the allele frequency data reported by the respective papers. We observed discrepancies between the two papers for 71 allele frequency distributions (Table S3). In one case, the cited source for a group did not reasonably match the file listed in the Supplement 2 of the aggregate study.^39^ No alternative source could be found, so we dropped this group from further analysis. Additional discrepancies between the aggregate and source allele frequency distributions include: individual frequency value differences (rounding to different degrees of precision, typos, values assigned to different alleles, values assigned to different groups reported by the same source), allele differences (dropped rare alleles, getting rid of alleles with a > or < designation, mislabeled alleles), and loci differences (loci reported in the aggregate table not seen in the source table) (Table S3). We eliminated groups with such discrepancies from our analysis if their genetic diversity values were typical, defined as being between 0.760 and 0.800 (Figure 1). Those discordant allele frequency tables for groups with extreme genetic diversity values (less than 0.760 or greater than 0.800) were replaced with the allele frequency tables from the source papers. We did this to preserve data from groups with unusual genetic diversity. This resulted in a total of 247 groups.The final quality control step checked that the frequencies across alleles for a locus sum between 0.99 and 1.01. This eliminated two groups from the aggregate study^39^ as well as one group from the FBI expanded dataset.^40^ After all our quality control steps, we have a set of 244 groups.

Upon review, we found that most source papers did not discuss obtaining informed consent from DNA sample donors. While all of those data remain publicly available, in the analysis presented here we only include data from papers that explicitly report informed consent. This leaves us with 83 groups in our analysis. The scripts used to evaluate the allele frequency tables and the final data tables employed in this study are posted at https://github.com/Forensic-Mixture-Analysis/ForensicMixtureAnalysis.

#### DNA mixture simulation approach

We used Forensim, a free open source R package,^38^ to simulate forensic genetic profiles of contributors, generate mixtures of those contributors, and compute likelihood ratios (LRs) to quantify the strength of evidence. To ensure that the simulated genotypes reflect provided reference allele frequency distributions, we compared the average expected heterozygosities (or genetic diversity) to observed heterozygosities in 100,000 simulated individuals per group (Figure S13). The average heterozygosity of simulated individuals closely approximates the genetic diversity for each group with a correlation of R^2^ = 0.9999.

LRs are computed as $LR=\frac{P\left(G\;|\;H_{p}\right)}{P\left(G\;|\;H_{d}\right)}$ where G is the genetic profile of the observed DNA mixture, H_p_ is the prosecution hypothesis that the POI contributed to the DNA mixture, and H_d_ is the defense hypothesis that the POI did not contribute to the DNA mixture, but rather an unknown individual contributed instead.^7^^,^^35^ These LRs take into consideration dropout probability parameters of 0.01 for heterozygotes and 0.0001 for homozygotes. When LR evaluates to <1 it indicates that the evidence is more likely under the defense hypothesis, and conversely, when it evaluates to >1, it indicates that the evidence is more likely under the prosecution hypothesis.^38^

Our study examines the accuracy of DNA mixture analysis assuming that the genotypes of all non-POI contributors in the simulated mixture are known under both the defense and prosecution hypotheses. While conditions for forensic mixture analysis may not typically conform to this ideal, it provides us an upper limit for estimating the accuracy.

In our early exploration of Forensim, we noticed on very rare occasions it would return different LRs given the same inputs. We address this with an *ad hoc.* computational fix by calculating all LRs twice and recalculating if the difference between the two LRs is greater than 0.001.

**Figure 7:**
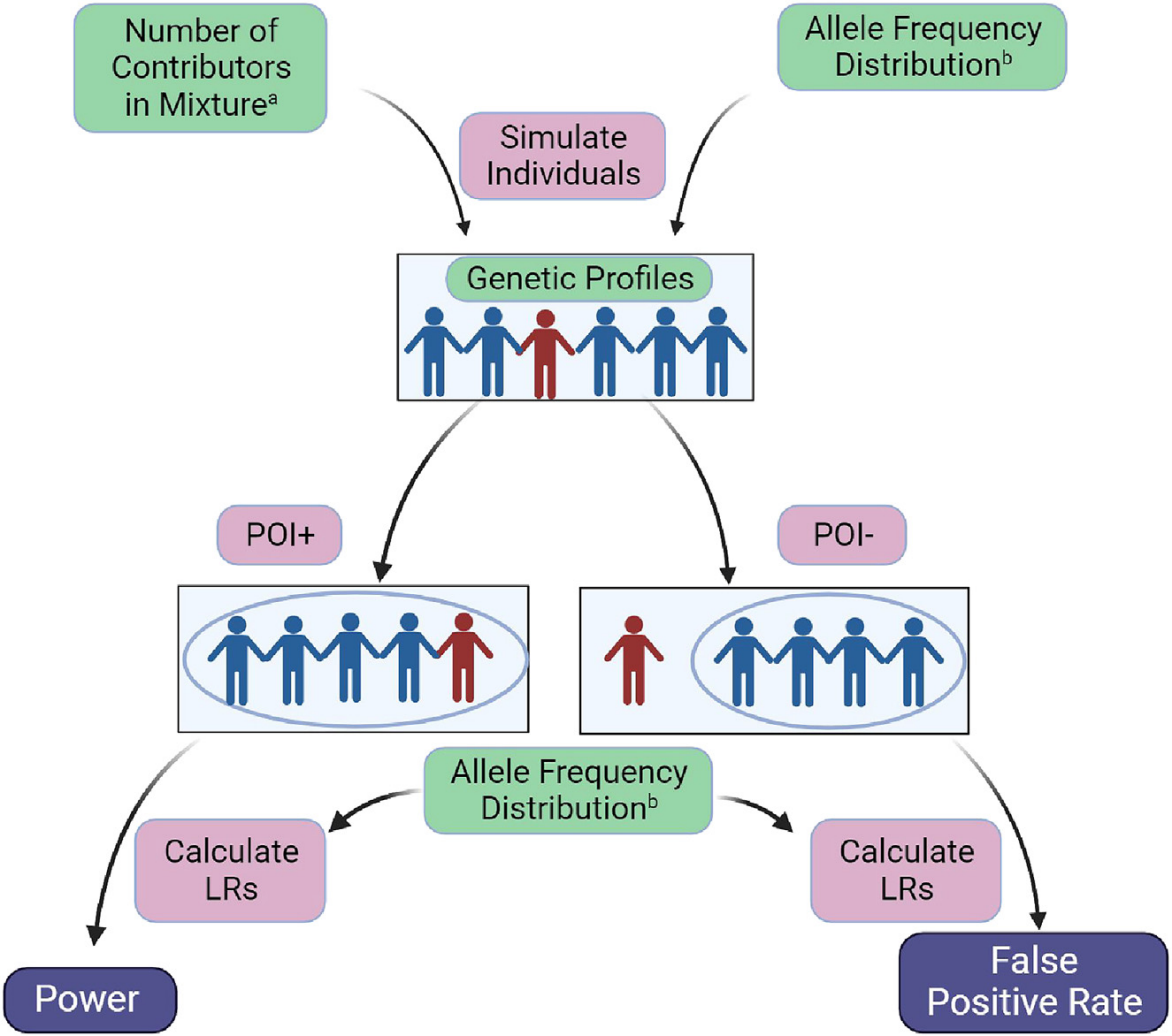
Simulating individuals, DNA mixtures, and calculating likelihood ratios with correctly specified reference groups This schematic shows how we varied the number of contributors to a mixture from two through six individuals^a^, as well as the allele frequency distribution to reflect each of the 83 reference groups^b^. With the resulting genetic profiles, we created two types of simulations: where the person of interest contributed (POI+), and where the person of interest did not contribute (POI-). We then used the same allele frequency distribution from the simulations to calculate LRs. We analyzed the results from POI+ mixtures to estimate power, and the results from POI- mixtures to estimate false positive rates.

We generated the individual DNA profiles and subsequent mixtures using a particular group’s allele frequency distribution. Our analysis examined mixtures ranging from two through six contributors; we simulated both when the POI is a contributor (POI+) to estimate power, and when the POI is not a contributor (POI-) to estimate false positive rates (FPRs). We repeated this analysis for each of the 83 groups. For each group, we simulated 100,000 mixtures, resulting in 1,000,000 LRs generated (two through six contributors, POI+ and POI-, 100,000 mixtures each) (Figure 7). We use a threshold of LR = 1 to determine if a mixture is interpreted to contain DNA from the POI (although see Figure S6 for results under alternative decision thresholds).

We assess accuracy using both FPR and power. FPR is the proportion of POI- mixtures with LR > 1. Power is the proportion of POI+ mixtures with LR > 1. Note that the false negative rate is 1 - *power*.

#### Mis-specified reference simulation approach

**Figure 8:**
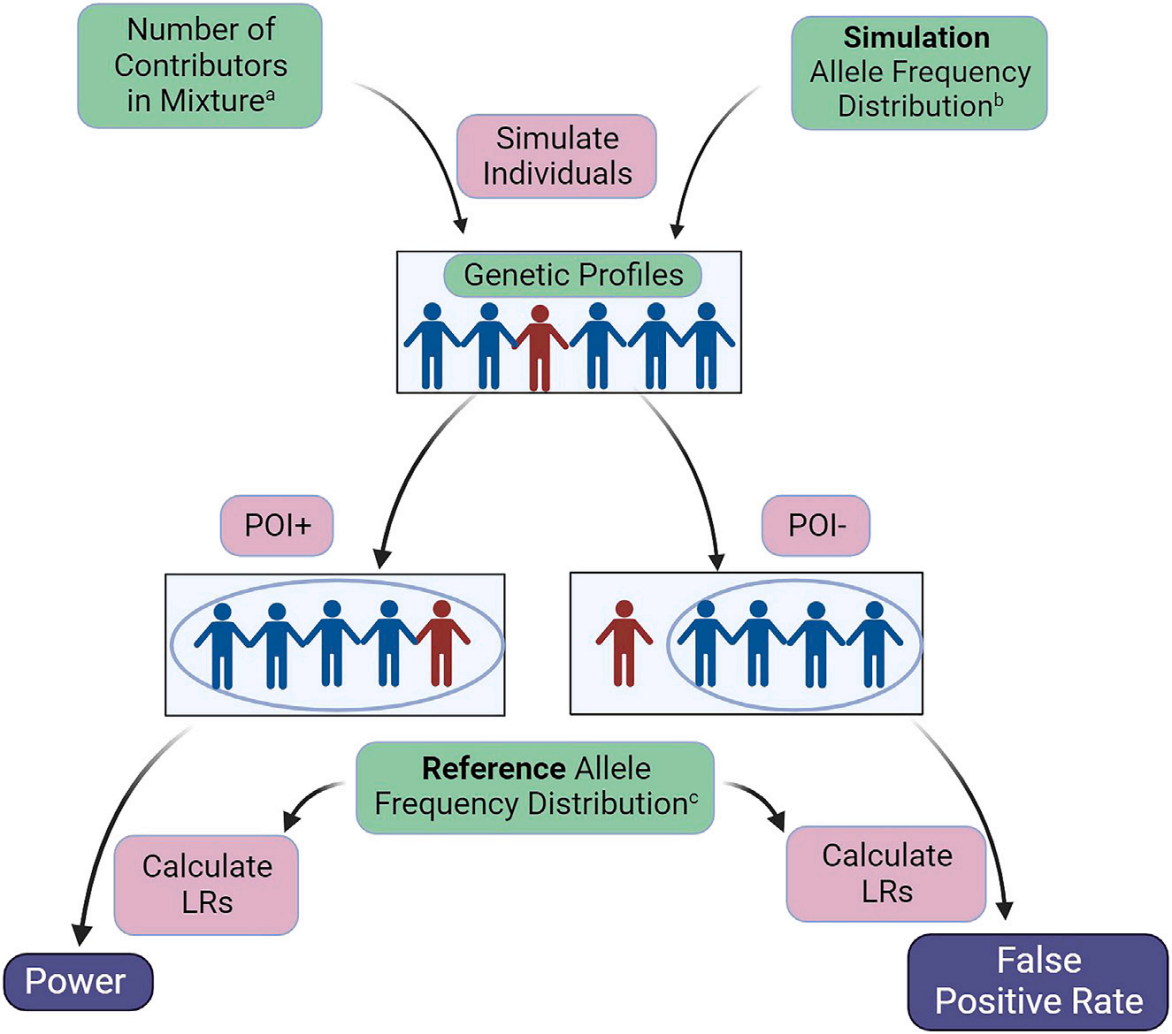
Simulating individuals, DNA mixtures, and calculating likelihood ratios with mis-specified reference groups This schematic shows how we varied the number of contributors in a mixture from two through six individuals^a^, as well as the simulation allele frequency distribution to reflect 26 groups^b^. Again, we created POI+ and POI- mixtures. We then used a different reference allele frequency distribution^c^ to calculate LRs. We analyzed the results from POI+ mixtures to estimate power, and the results from POI- mixtures to estimate false positive rates.

To estimate the accuracy of DNA mixture analysis when an inappropriate reference allele frequency distribution was used, we performed the analysis where one allele frequency distribution is used to simulate individuals (the simulation allele frequency distribution), while a different allele frequency distribution is used to compute the LR (the reference allele frequency distribution) (Figure 8). Apart from this, the structure of the analysis remains similar to the previous analysis. For each pair of simulation and reference groups, we varied the number of contributors from two through six individuals. We simulated POI+ and POI- mixtures to estimate power and FPRs respectively. Again, we use a threshold of LR = 1 to decide if a mixture is interpreted to contain DNA from the POI.

Because this analysis is computationally intensive (O(n^2^) with the number of groups), it was performed on 26 out of the 83 groups (see subsection “Subsetting for computational efficiency”). For each ordered pair of those groups, we simulated 10,000 mixtures with one group as reference and the other for simulations, resulting in 100,000 LRs (two through six contributors, POI+ and POI-, 10,000 mixtures each) per ordered pair. That is, among the 26 groups described above, we considered every possible combination of simulation and reference groups.

#### Subsetting for computational efficiency

It is computationally infeasible to vary both the simulation and reference groups over such a large list of 83 groups. In order to address this, we trimmed down the number of groups used. We identified genetically similar groups based on their F_ST_ and removed one of them. This left us with a smaller set of genetically diverse groups.

Specifically, we calculated F_ST_ between each pair of groups using the method by Weir and Cockerham,^111^ which accounts for groups of different sizes, and across multiple alleles and loci. We then iteratively identified the pair of groups with the lowest F_ST_ and randomly removed one of those two groups, repeating until all F_ST_ values exceed a threshold. For this analysis, we used a threshold F_ST_ value of 0.005.

In the end, 26 groups remained, encompassing a genetic diversity range of 0.68–0.80 (Figure S1). Note that the FBI expanded groups^40^ and the groups reported by Hill et al.^85^ did not undergo filtering based on F_ST_ value because their allele frequency distributions are widely used by the field.^40^^,^^85^

### Quantification and statistical analysis

Analysis of significance was done using Pearson correlation test in R. Significance levels (P-values) are indicated in the results.
