# Supplementary material for: Decreased accuracy of forensic DNA mixture analysis for groups with lower genetic diversity
Source: iScience. 2024 Sep 28;27(11):111067. doi: 10.1016/j.isci.2024.111067 (PMC11539586; doi:10.1016/j.isci.2024.111067)
Supplement: Document S1. Figures S1–S13 and Table S1 [file mmc1.pdf]

iScience, Volume *27*

Supplemental information

Decreased accuracy of forensic DNA mixture analysis for groups with lower genetic diversity

Maria Flores, Evan Ho, Cara Ly, Niquo Ceberio, Miguel Guardado, Kamillah Felix, Hannah Mariko Thorner, Matt Paunovich, Chris Godek, Carina Kalaydjian, and Rori V. Rohlfs

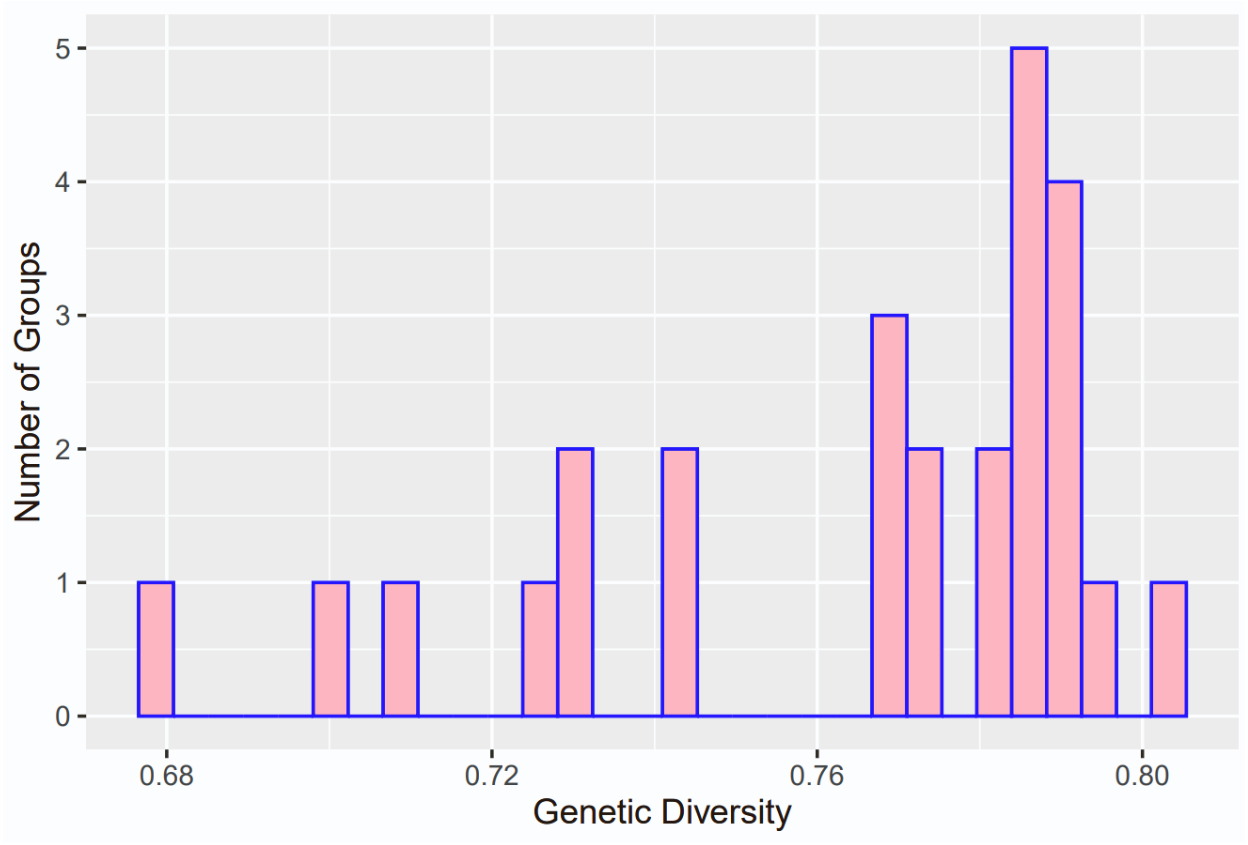

Figure S1: Distribution of genetic diversity over the subset of genetically distinct groups, related to Figure 1. Genetic diversity of 26 groups analyzed for the accuracy of DNA mixture analysis with a misspecified reference.

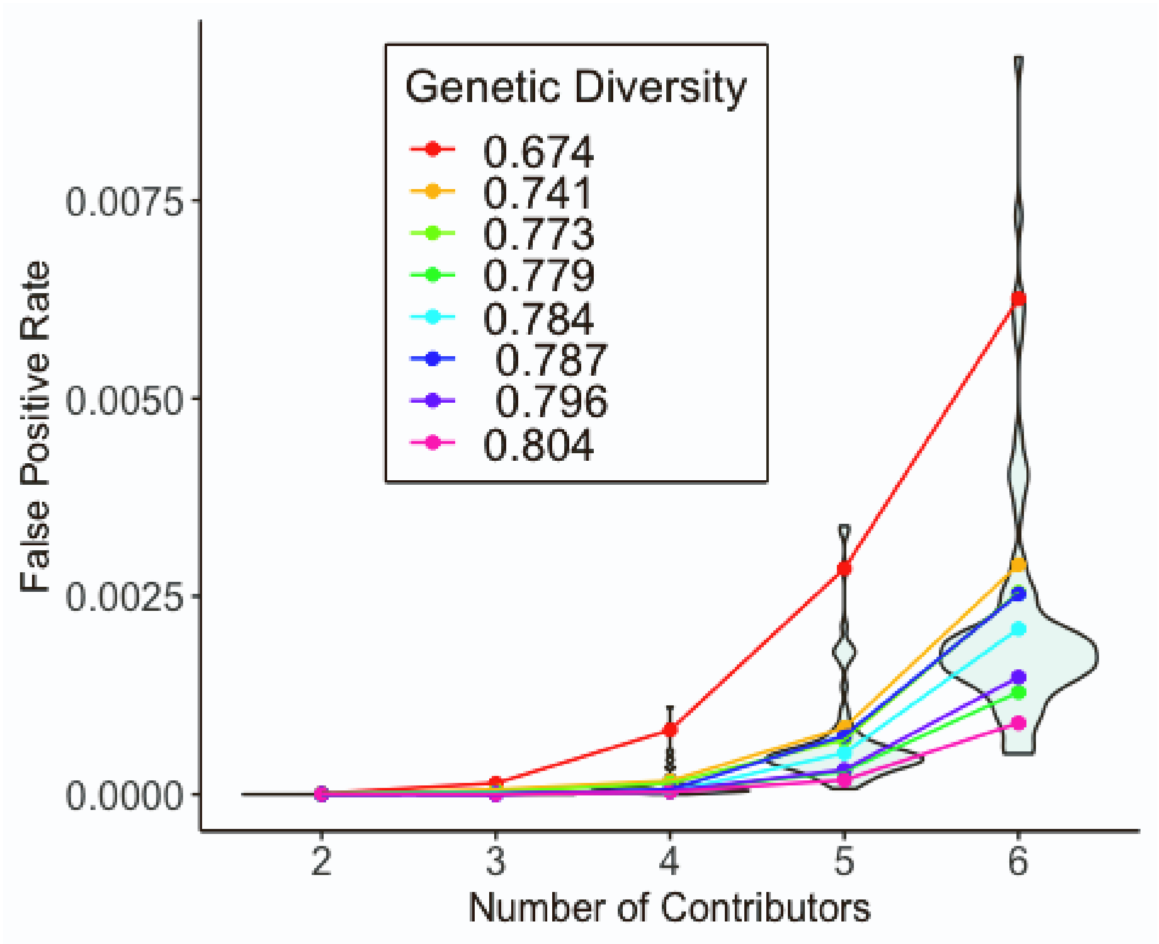

Figure S2: False positive rates (linear scale y-axis) distributions when reference group is correctly specified (prDHet = 0.01 and prDHom = 0.0001), related to Figure 2. False positive rates are shown for some specific groups, representing quantiles of genetic diversity.

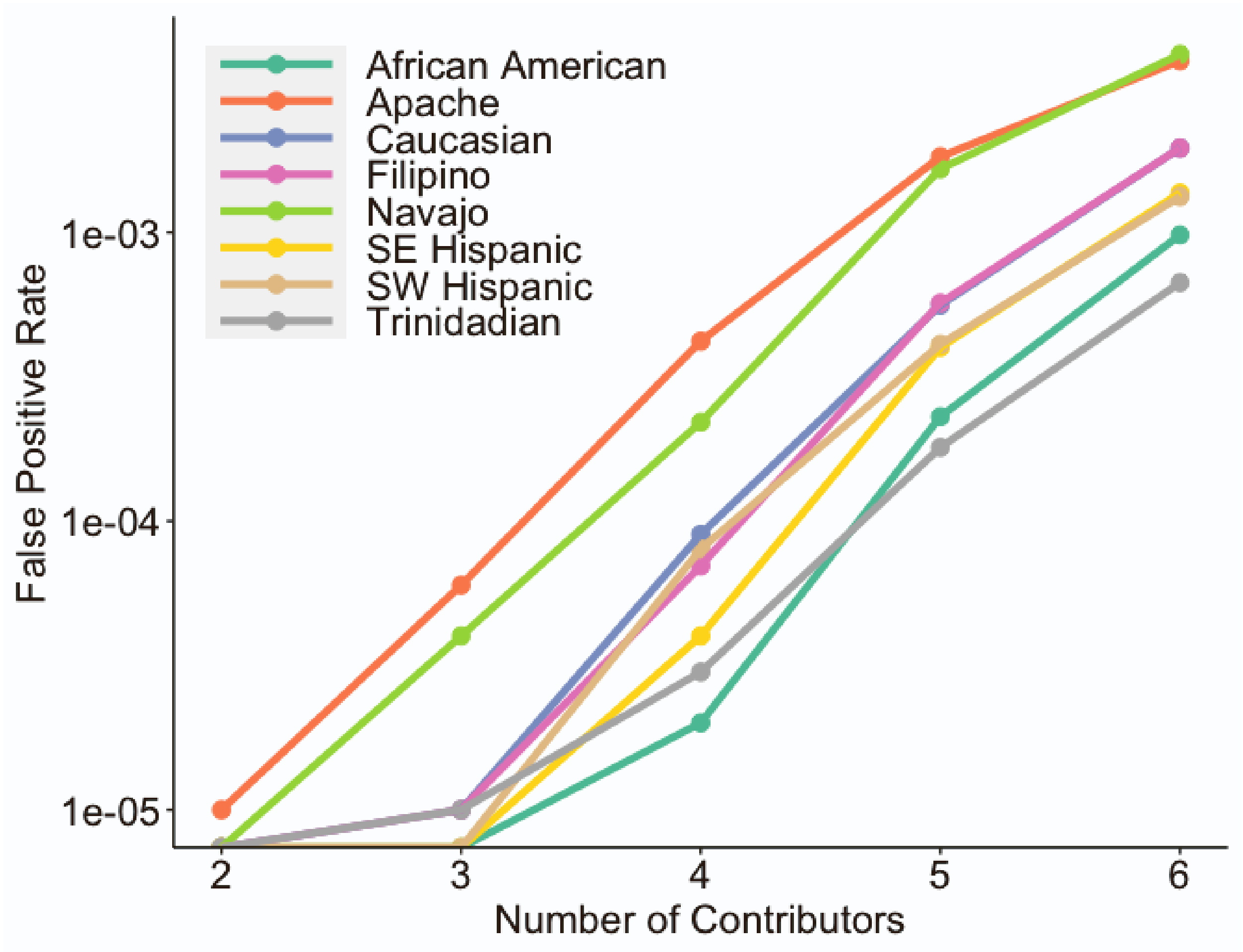

Figure S3: False positive rates across FBI reference groups (prDHet = 0.01 and prDHom = 0.0001), related to Figure 2. Note that the groups are labeled with the original descriptors[S1].

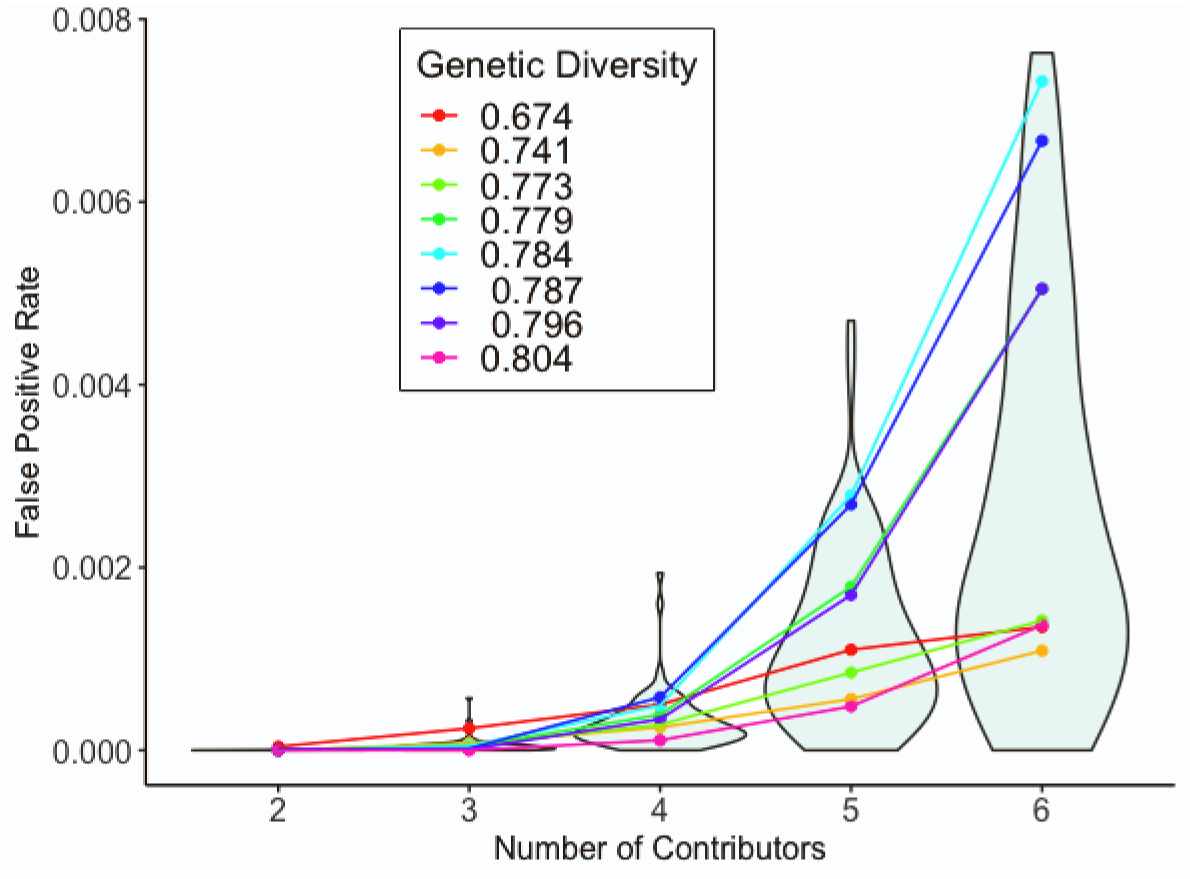

Figure S4: False positive rates (linear y-scale axis) distributions when reference group is correctly specified and when dropout rate parameters for LR calculations are increased (prDHet = 0.02 and prDHom = 0.04), related to Figure 2. False positive rates are shown for some groups, representing quantiles according to genetic diversity.

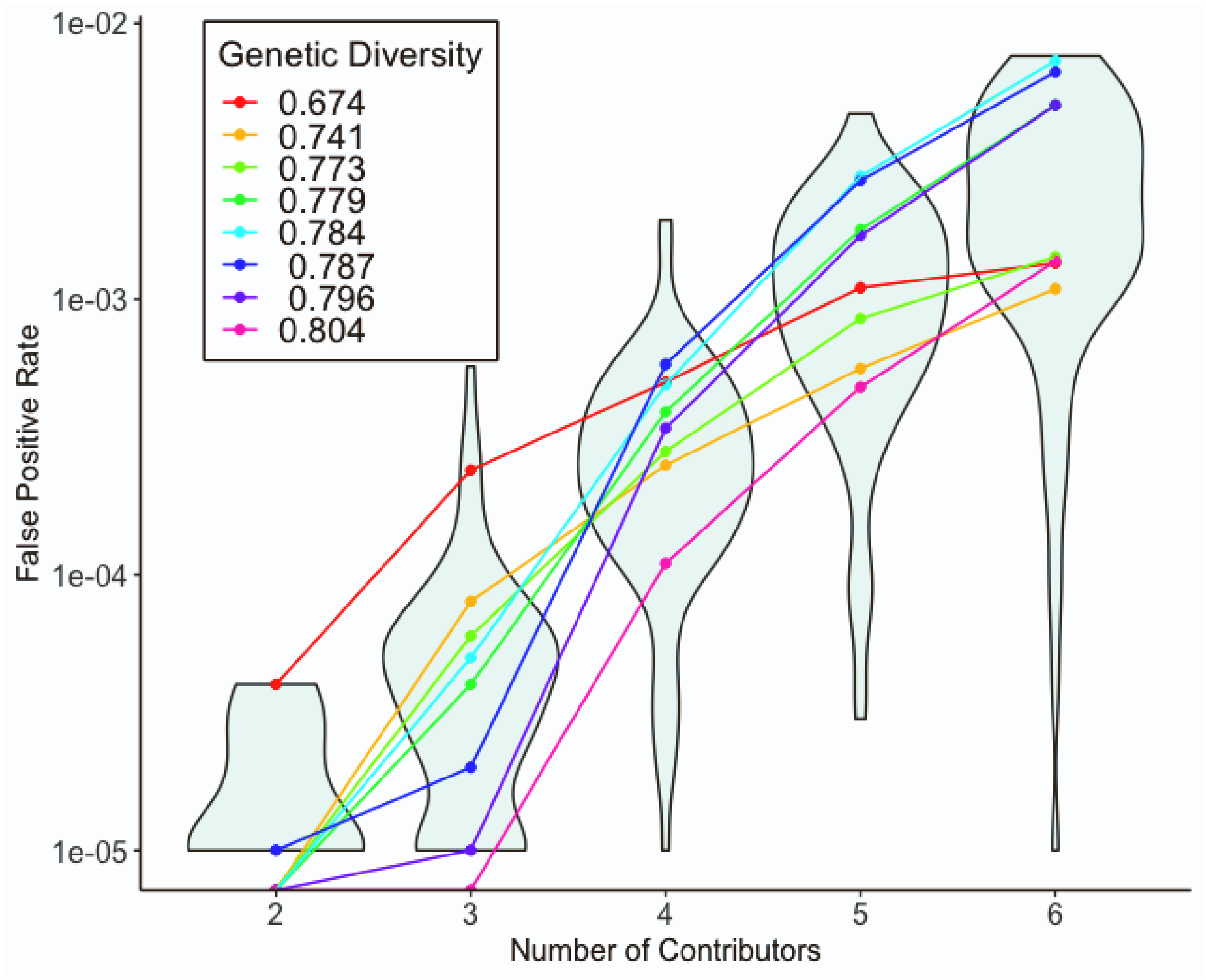

Figure S5: False positive rates (log scale y-axis) distributions when reference group is correctly specified and dropout rate parameters for LR calculations are increased (prDHet = 0.02 and prDHom = 0.04), related to Figure 2. False positive rates are shown for some groups, representing quantiles according to genetic diversity.

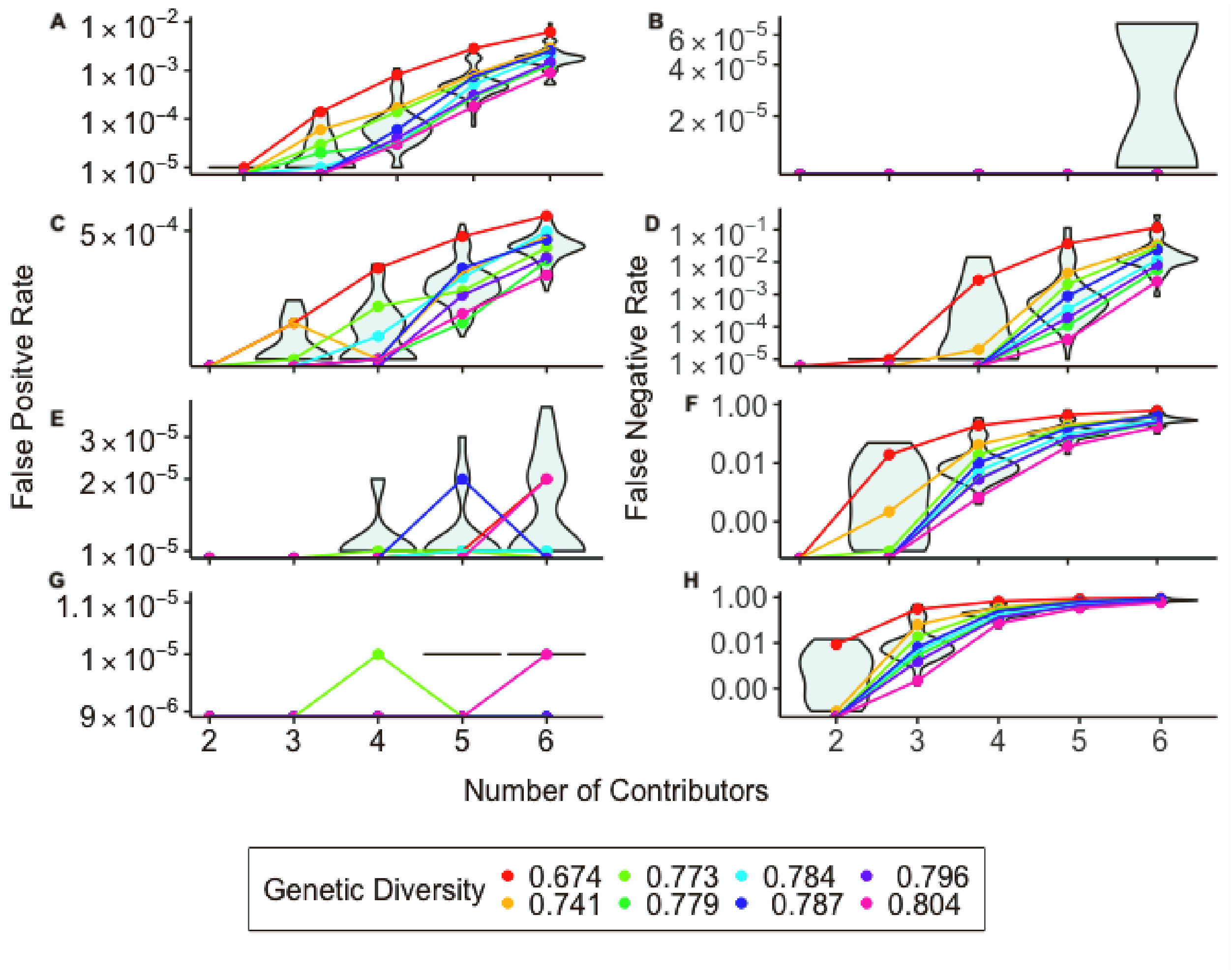

Figure S6: False positive and false negative rates when the reference group is correctly specified and under varying decision thresholds (prDHet = 0.01 and prDHom = 0.0001), related to Figure 2. False positive rates when LR decision threshold is greater than (A) 1 (C) 100, (E) 10,000 and (G) 1,000,000. False negative rates when LR decision threshold is greater than (B) 1, (D) 100, (F) 10,000 (H)1,000,000. False positive and false negative rates are shown for groups representing the quantiles according to genetic diversity. Note that the y-axes are in log scale.

Table S1: Power estimates from the 83 groups that did not equal 1, related to Accuracy of DNA mixture analyses decreases with genetic diversity results.

Genetic Diversity	Power Rate	Number of Contributors
0.724	0.99993	6
0.6781	0.99999	6

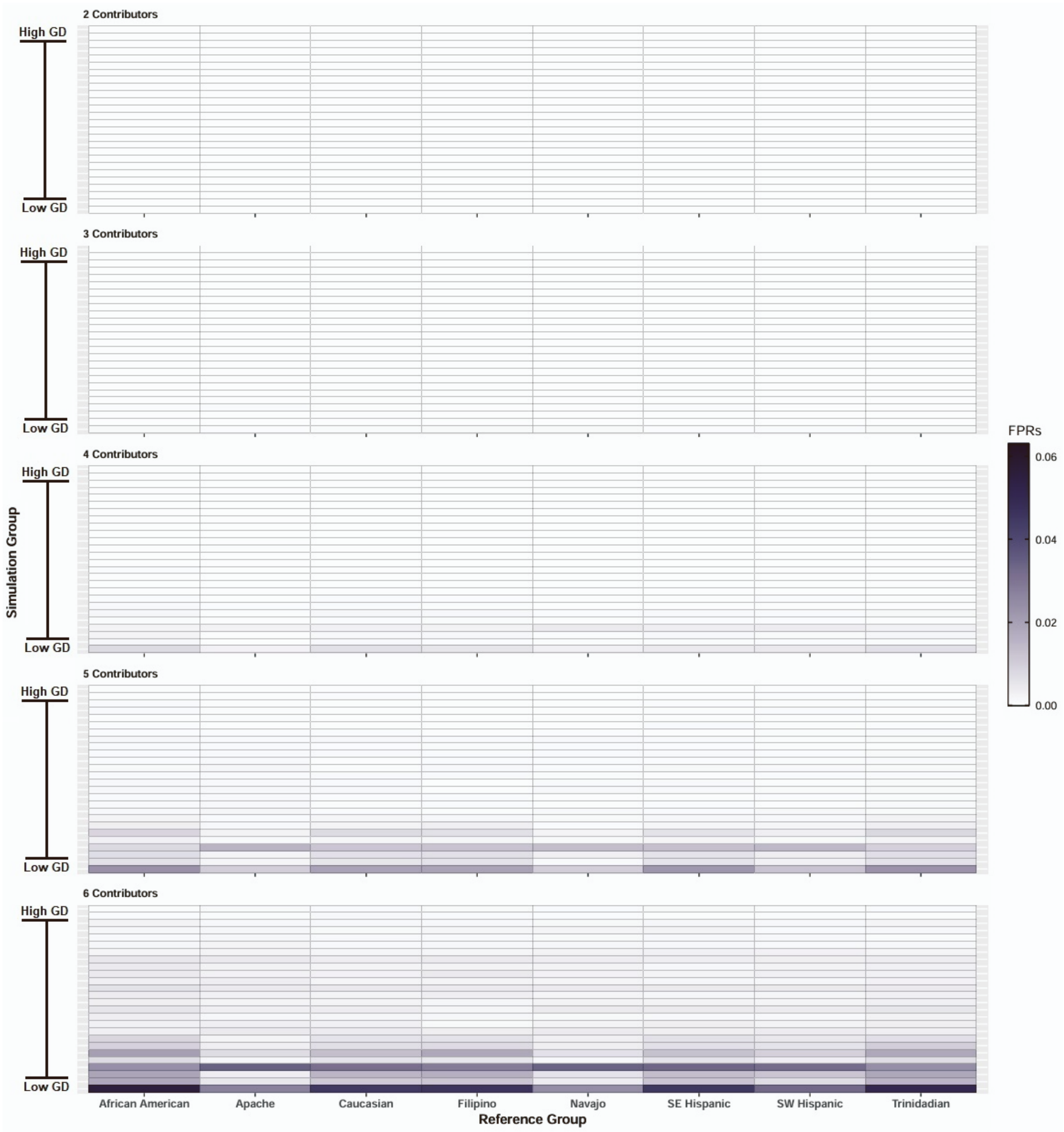

Figure S7: False positive identification rates with FBI reference groups, related to Figure 4. False positive rates are shown for analyses with 26 simulation groups and 8 of the FBI reference groups[S1]. Simulation groups are arranged from low to high genetic diversity, and mixtures ranging from two through six contributors were analyzed.

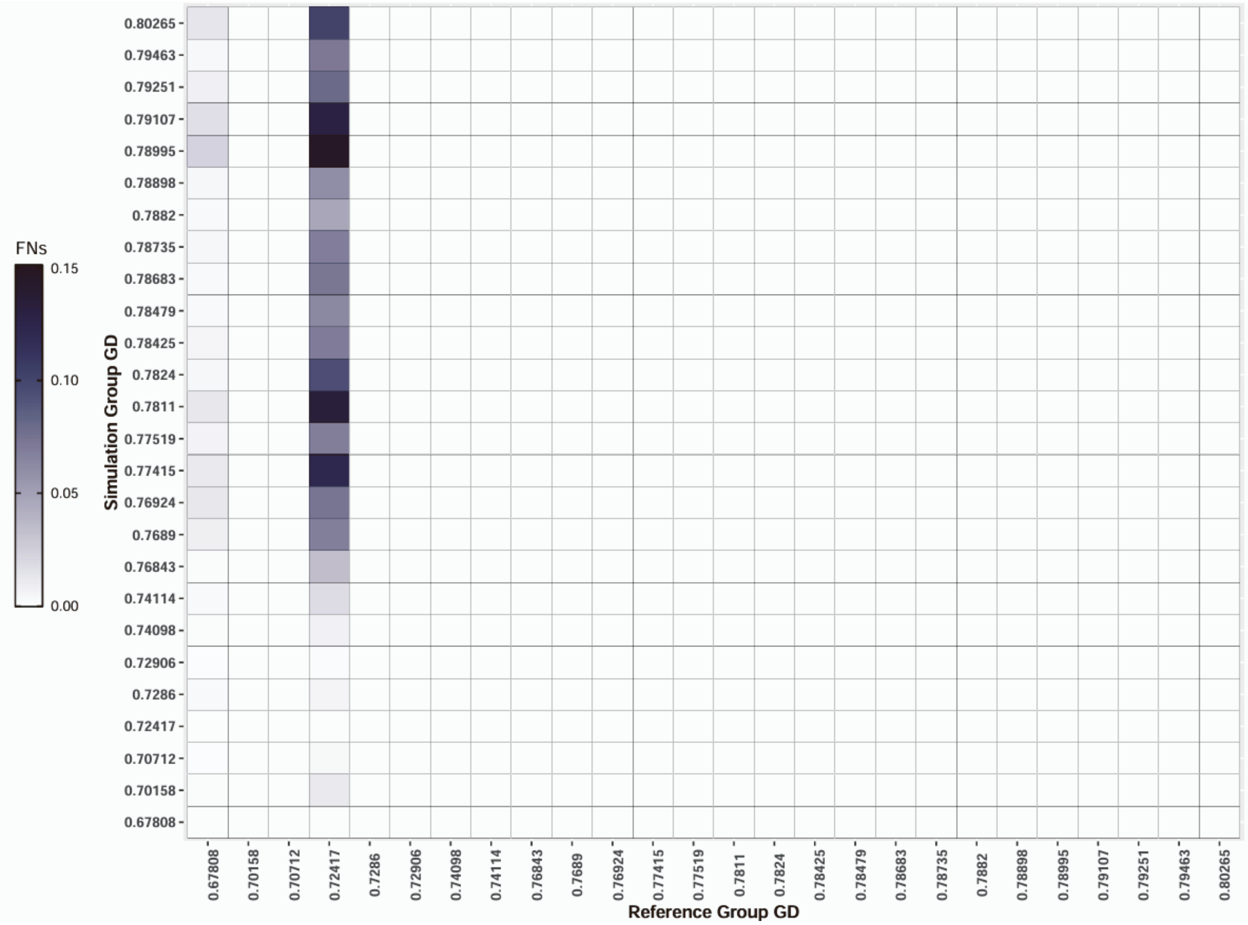

Figure S8: False negative identification rates with correctly and incorrectly specified reference groups, related to Figure 5. The groups are arranged by genetic diversity on the axes. The false positive rates are for the analysis of six-contributor mixtures. Note that the diagonal represents correctly specified reference groups, and that false negatives only occur when 0.67808 and 0.72417 genetic diversity groups are used as the reference group.

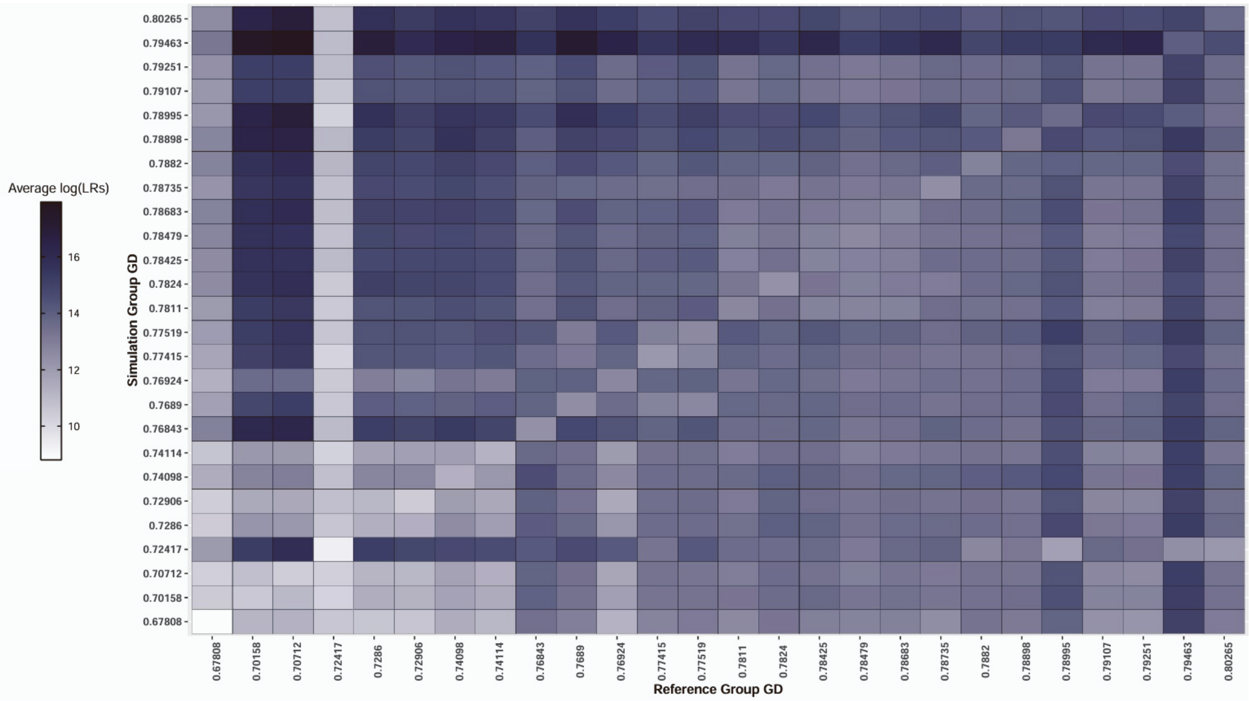

Figure S9: POI+ Average log(LRs) with correctly and incorrectly specified reference groups, related to Figure 5. The groups are arranged by genetic diversity on the axes, and this analysis is for six-contributor mixtures. Note that the diagonal represents correctly specified reference groups.

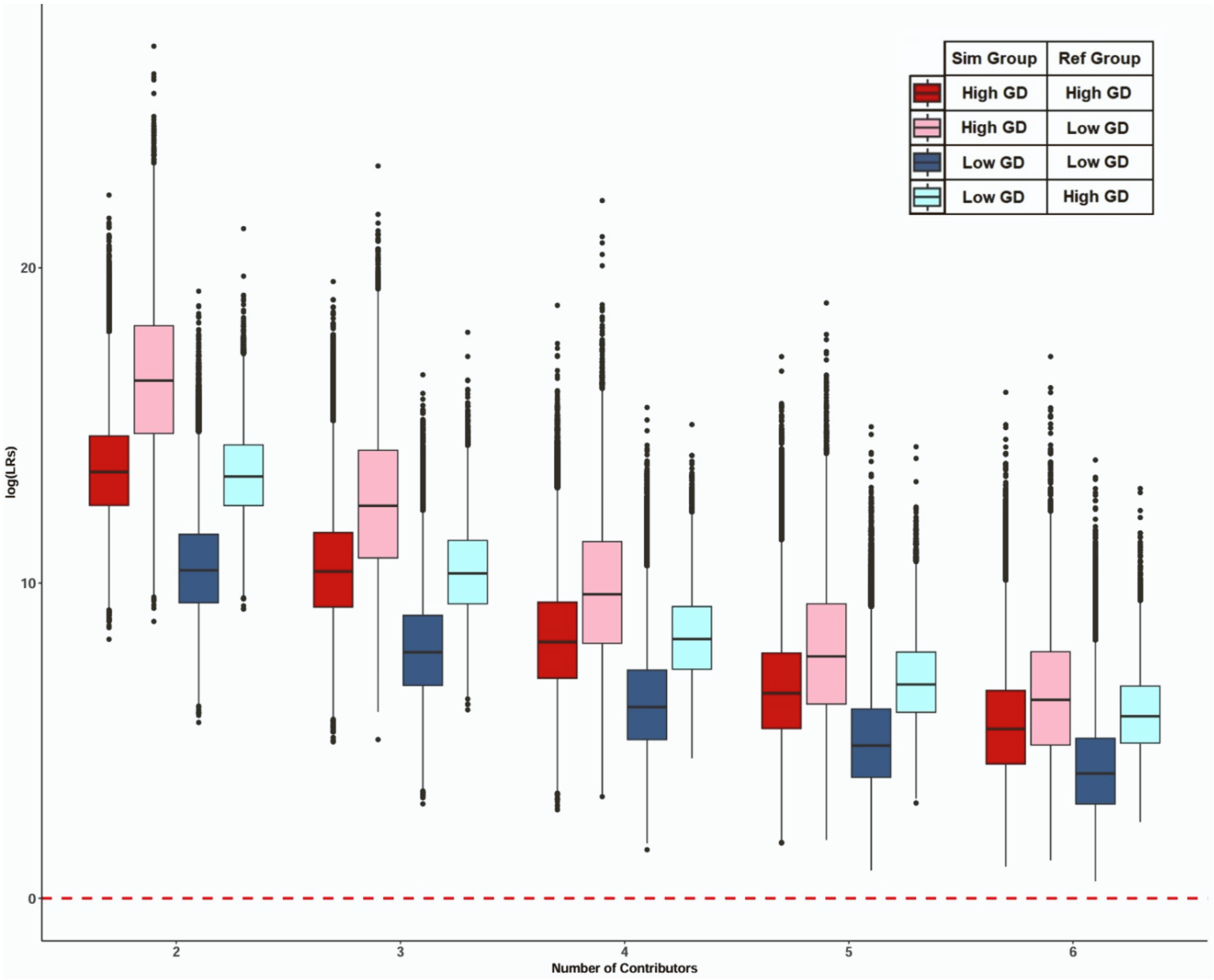

Figure S10: Distributions of log(LR) for POI+ mixtures with correct and misspecified reference groups, related to Figure 5. This plot compares the distributions of log(LR) for POI+ mixtures for high (0.80) and low (0.70) genetic diversity (GD) groups, with accurately specified and misspecified reference groups, over two through six contributors.

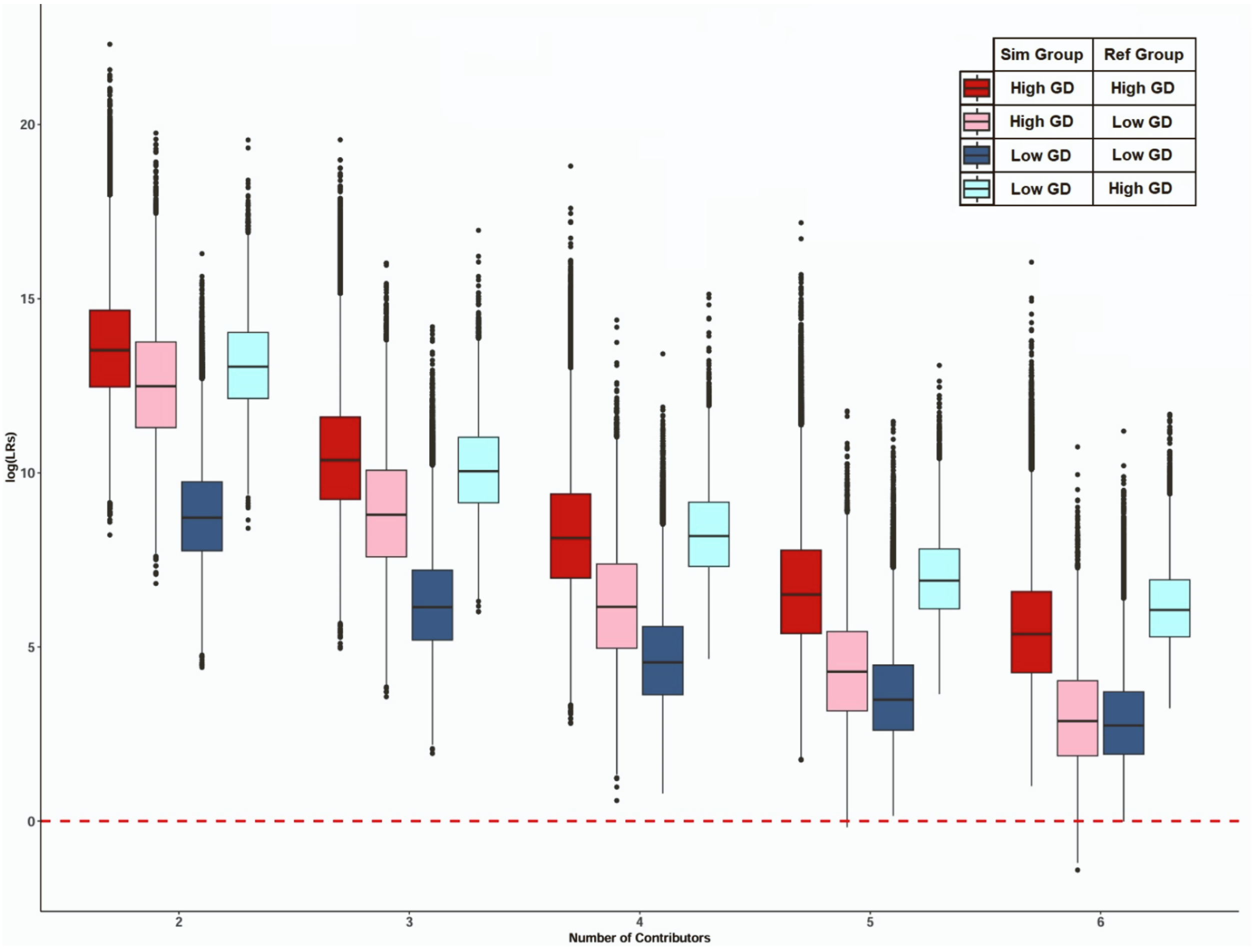

Figure S11: Distributions of log(LR) for POI+ mixtures for the highest and lowest genetic diversity groups, related to Figure 5. This plot compares the distributions of log(LR) for POI+ mixtures for the highest (0.80) and lowest (0.68) genetic diversity (GD) groups from our subsetted group list, with accurately specified and misspecified reference groups, over two through six contributors. Log(LR) values that fall below 0 are false negatives.

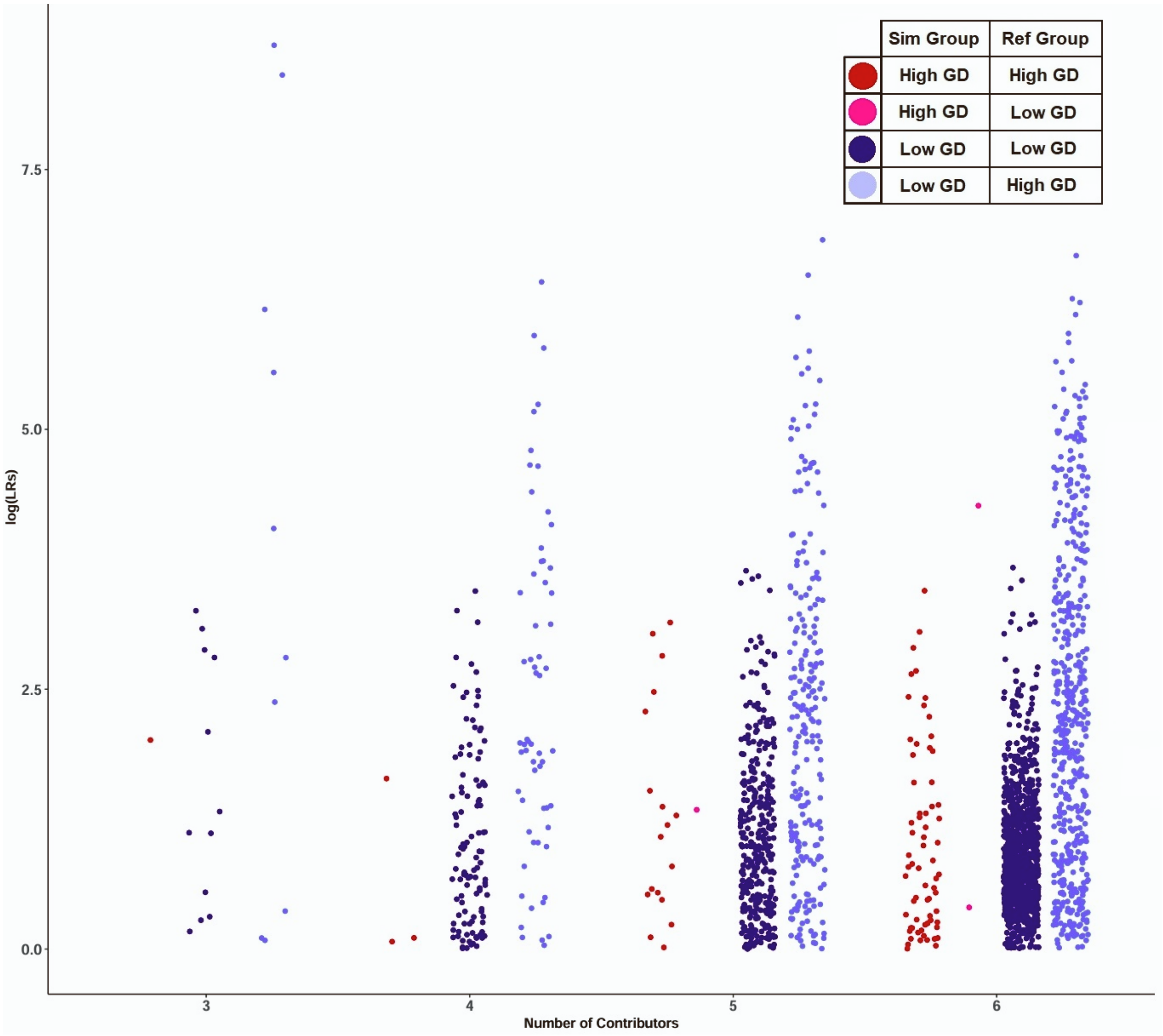

Figure S12: POI- log(LR) distributions with correct and mis-specified reference groups, of the highest and lowest genetic diversity groups, related to Figure 5. This plot compares the distributions of log(LR) for POI- mixtures for high (0.80) and low (0.68) genetic diversity (GD) groups.The log(LR) distributions for POI- analyses are shown across two through six contributor mixtures. The darker blue and red distributions show when reference groups are correctly specified, for low and high GD groups, respectively. The lighter blue and pink show when reference groups are misspecified, for low and high GD simulation groups, respectively. Note that there are no false positives for two contributor mixtures.

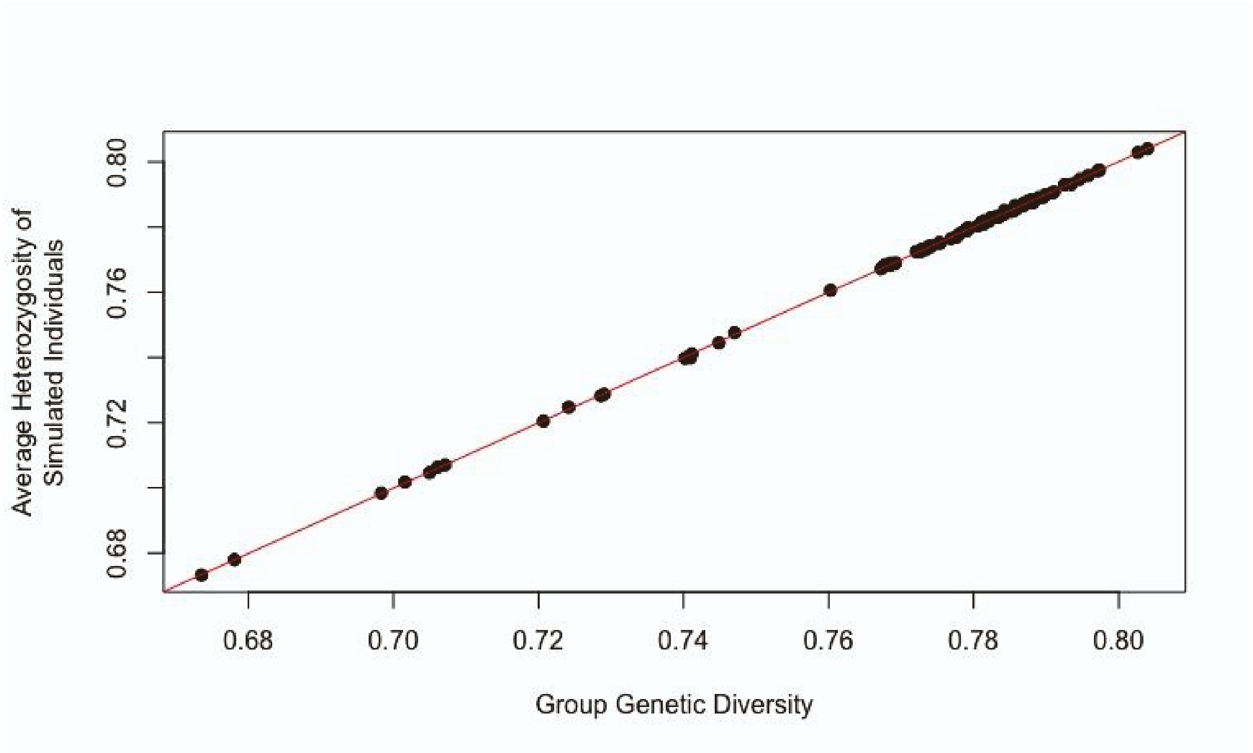

Figure S13: Comparison of expected and simulated average heterozygosities, related to Figure 1. This plot compares the genetic diversity (or average expected heterozygosities) to the observed average heterozygosities for simulated individuals. Results are shown for the 83 groups analyzed in this manuscript.

Table S2: Power estimates from the 26 mis-specified reference groups that did not equal 1, related to Accuracy of DNA mixture analysis decreases with mis-specified reference allele frequency distribution.

Table S3: Previously published group labels and their genetic diversities, related to STAR Methods.[S2]

Table S4: Allele frequency table discrepancies observed, related to STAR Methods.[S2],[S3]

References:

[S1]. Steffen, C.R., Coble, M.D., Gettings, K.B., and Vallone, P.M. (2017). Corrigendum to ‘U.S. Population Data for 29 Autosomal STR Loci’ [Forensic Sci. Int. Genet. 7 (2013) e82–e83]. Forensic Sci. Int. Genet. *31*, e36–e40. 10.1016/j.fsigen.2017.08.011.

[S2]. Buckleton, J., Curran, J., Goudet, J., Taylor, D., Thiery, A., and Weir, B.S. (2016). Population-specific F values for forensic STR markers: A worldwide survey. Forensic Sci. Int. Genet. *23*, 91–100. 10.1016/j.fsigen.2016.03.004.

[S3]. Ruitberg, C.M. (2001). STRBase: a short tandem repeat DNA database for the human identity testing community. Nucleic Acids Res. *29*, 320–322. 10.1093/nar/29.1.320.
